# Changes in health behaviour of medical students during and after the COVID-19 pandemic—focus on physical activity, screen time, sleep duration, unhealthy foods, alcohol, and tobacco

**DOI:** 10.3389/fpubh.2025.1545295

**Published:** 2025-03-24

**Authors:** Lukas Liebig, Willy Gräfe, Hendrik Berth, Béla Birkás, Nora Faubl, Erika Zelko, Erika Balogh, Henna Riemenschneider

**Affiliations:** ^1^Department of General Practice, Faculty of Medicine Carl Gustav Carus, TUD Dresden University of Technology, Dresden, Germany; ^2^Research Group Applied Medical Psychology and Medical Sociology, Division of Psychosocial Medicine and Developmental Neurosciences, Faculty of Medicine Carl Gustav Carus, TUD Dresden University of Technology, Dresden, Germany; ^3^Department of Behavioral Sciences, University of Pécs Medical School, Pécs, Hungary; ^4^Institute of General Practice, Johannes Kepler University Linz, Linz, Austria; ^5^Department of Public Health Medicine, University of Pécs Medical School, Pécs, Hungary

**Keywords:** medical students, COVID-19 pandemic, health behaviour, physical activity, screen time, sleep, alcohol, tobacco

## Abstract

**Introduction:**

The COVID-19 pandemic and the preventive measures led to a change in the health behaviour among the population. Medical students were particularly affected by this. Previous studies primarily focused on few health behaviours, were mostly conducted in 2020–2021, and did not assess the persistence of these behaviours post-pandemic.

**Methods:**

A combined cross-sectional and longitudinal approach were applied to examine changes in physical activity, screen time, sleep duration and consumption of unhealthy foods, alcohol and of tobacco. Data from Medical Students at the Technical University of Dresden were collected online as part of the multicenter study “Medical Student Health Survey” in 2020 and 2022. Descriptive and inferential statistical methods were applied.

**Results:**

Medical students (*N* = 575) reported reduced physical activity and increased screen time due to the COVID-19 pandemic, citing lockdown and changed habits as main reasons. Longitudinal analysis of medical students (*N* = 66) between 2020 and 2022 revealed increased physical activity (*p* = 0.018) and decreased unhealthy food consumption (*p* = 0.009) after the end of the pandemic. Screen time, sleep duration and consumption of alcohol and of tobacco products remained unchanged. Changes in health behaviours were not intercorrelated.

**Discussion:**

The COVID-19 pandemic led to change in health behaviour of medical students. However, intra-pandemic changes differing from pre-post pandemic changes and interindividual variations in health behaviour change were found. The rise in physical activity, the decreased consumption of unhealthy foods, and the low tobacco use reflect a health-aware cohort. The findings should inform the development of future preventive measures and further research is needed to understand the sustainability and broader impact of these health behaviour changes.

## Background

During the COVID-19 pandemic, the measures adopted to contain the COVID-19 virus led to a change in the lifestyles of various population groups. Harmful health behaviour in particular (1, 2) and psychosocial phenomena such as stress (3) increased as a result of measures such as the closure of public buildings and educational institutions, as well as contact bans. In addition to the older population groups, young people in particular reportedly suffered from the consequences of the COVID-19 pandemic (4, 5).

Medical students were also affected by the impact of the COVID-19 pandemic. The sudden switch to online teaching and examinations posed a challenge for medical students, as their studies are include practical experience and laboratory and clinical work (6, 7). In addition, many medical students were directly involved in managing the pandemic, whether through supporting activities in hospitals or working in testing centres. The associated concerns about their own health and that of their patients were a source of additional stress (7).

There is already empirical evidence, which indicates that the COVID-19 pandemic has significantly influenced health behaviour of students (8–10) and especially medical students (11–13). However, these studies focused on individual aspects of health behaviour (such as nutritional behaviour or mental health) and were conducted in a cross-sectional design. In addition, previous studies are mostly from the years 2020–2021 and cannot provide any insight into the extent to which certain behaviours remain prevalent after the end of the pandemic.

This study aims to close this research gap and provide an overview of the changes in the health behaviour of medical students in Germany from the beginning to the end of the COVID-19 pandemic. In order to provide an overview of various health-relevant behaviours, the following aspects are examined: physical activity, screen time, sleep duration, as well as consumption of unhealthy foods, alcohol and of tobacco products. In addition, this study combines a cross-sectional analysis (2022), subsequent Sub-study A, with a longitudinal analysis (2020–2022), subsequent Sub-study B.

The study will reveal trends in medical students’ health behaviour, highlighting the impact of COVID-19 measures on medical students’ health behaviour and informing the development of new prevention strategies.

## Methods

### Study protocol

The Department of General Practice at the Carl Gustav Carus Faculty of Medicine, Technical University Dresden (TUD), Germany conducted cross-sectional multi-center study “Medical Student Health Survey” (MSHS) in 2020 and 2022, as a collaboration with the Research Group for Applied Medical Psychology and Medical Sociology at the Carl Gustav Carus University Hospital, TUD; the Departments of Public Health Medicine and Behavioral Sciences at the University of Pécs, Hungary; and, in 2022, the Institute of General Medicine at the Johannes Kepler University Linz, Austria. This study focused on the data from medical students in Dresden (Germany), as the preventive measures implemented during the pandemic varied significantly in timing and content across study locations.

**Figure 1:**
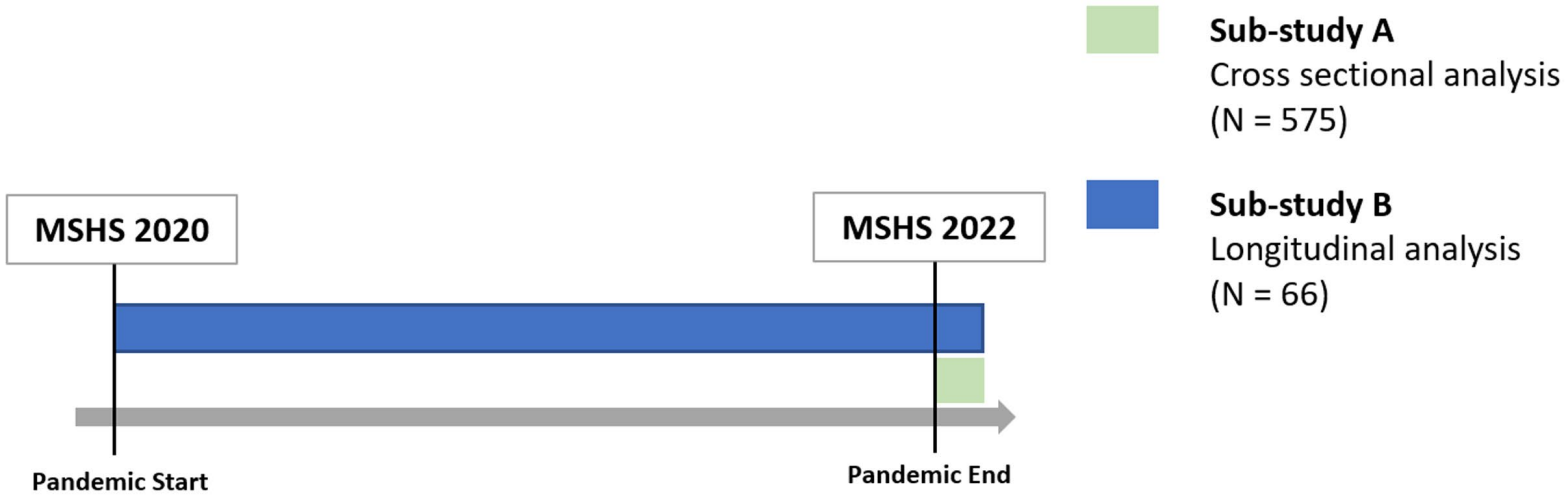
Study structure: Sub-study A and Sub-study B.

The medical students at the TUD were invited to participate voluntarily and anonymously. Written information was provided in advance and informed consent to data processing was obtained. Invitations to participate in the online survey were sent via various channels such as email invitations from the Medical Student Council and the Teaching Department at the TUD Faculty of Medicine, digital advertising on social media and active recruitment during lectures and seminars. The survey period ran from January to July 2020 in 2020 and from June to October 2022 in 2022. In 2020, the first preventive measures (lockdown, online teaching) were imposed in mid-March and lasted until the beginning of May, during which time the survey was paused to minimise the potential impact of these measures. In 2022, the only measures still in place at the start of the survey were the obligation to wear masks on local public transport and in hospitals and doctors’ surgeries. Measures such as lockdowns or a ban on face-to-face teaching no longer applied at this time and have not been enacted since. As a result, the 2020 survey represents the beginning of the pandemic and the survey in 2022 represents the end of the pandemic in the context of this work (see Figure 1).

### Measurements

The online questionnaire (applied via LimeSurvey version 2.50+) was compiled from standardised and specially developed questions and covered 9 topics. In addition to socio-demographic questions, the health status (general and mental health), health behaviour such as sleeping, eating and exercise habits and media consumption were recorded. In 2022, health behaviour change due to the COVID-19 pandemic was also surveyed. Each questionnaire included the voluntary provision of an individual pseudonymisation code, which makes it possible to compare a person’s data over several years.

The subject of Sub-study A was the analysis of the self-assessment recorded in MSHS 2022 on changes in health behaviour due to the COVID-19 pandemic. Information was provided for (1) physical activity, (2) screen time, (3) sleep duration, as well as (4) consumption of unhealthy foods, (5) alcohol and (6) tobacco products. For the following analysis, the responses were summarised in “decrease,” “no change,” “increase” to ensure clarity and distinct differentiation between change and no change. If the students indicated a change, they were given the opportunity to state the reasons why their health behaviour had changed. The answer options (multiple answers possible) were: changed habits, Lockdown/regulations, fear/worries, financial reasons, time reasons, motivational reasons, health-related reasons, other. All items including answer options of Sub-study A are shown in Supplementary material 1.

The subject of Sub-study B was the longitudinal analysis of the information on the health behaviour of those students who took part in the Medical Student Health Survey 2020 and 2022 and were identifiable via a pseudonymisation code. The items of the respective years (2020–2022) for recording health behaviour (1–6) and the scoring system are shown in Supplementary material 2.

In order to ensure comparability between study population A and B, socio-demographic data (age, gender) and socio-economic factors such as the study period, the existence of a relationship or children, the housing situation and existence of financial problems were also recorded. The questions on the existence of a relationship and own children could be answered with yes or no. The other information was also dichotomised: study period into preclinical (1st–4th semester) and clinical (≥5th semester), housing situation into living alone vs. not living alone, and financial problems (Likert Scale from 1 to 5) into no financial problems (1-2) vs. existing problems (3-5).

### Statistical analysis

The statistical analyses were carried out using IBM SPSS 28.0. For Sub-study A, the information provided by the students was analysed descriptively. For Sub-study B, sum scores were calculated in accordance with Supplementary material 1. The data were tested for normal distribution using the Shapiro–Wilk test. Medians of non-normally distributed variables were tested for significance using the Wilcoxon sign test for paired samples if symmetry was given. If there was no symmetry in the data, the sign test for paired samples was used. Differences in the longitudinal section in dichotomous data were tested for significance using the exact Mc Nemar test. A significance level of *p* ≤ 0.05 was used. No prior performance analysis was conducted due to the hypothesis-generating nature of the study.

### *Post hoc* analysis

Based on the results of Sub-study B, it was decided to carry out a *post hoc* analysis. The aim was to explore whether a change in one health behaviour (for example: increased physical activity) was associated with a change in another health behaviour (for example: less alcohol consumption). For this purpose, the respective individual changes in the sum scores (2022–2020) were checked analysed for correlation using a non-parametric test (Kendal’s tau-B).

### Ethical vote

This study was conducted in accordance with the principles of the Declaration of Helsinki. Approval was granted by the Ethics Committee of the Technical University of Dresden (No. EK 15012014).

## Results

### Study population

#### Sub-study A

With *N* = 720 completed questionnaires from *N* = 1986 enrolled medical students at the TUD, the response rate in 2022 was approx. 36%. Of the *N* = 720 questionnaires, only those students were included (*N* = 575), who provided information on changes in health behaviour due to the COVID-19 pandemic. The study population was *M* = 24.06 years old (SD = 3.9) and 71.5% were female (see Table 1).

**Table 1:** Study populations of Sub-study A and Sub-study B.

	Sub-study A (2022)	Sub-study B (2020–2022)
Total *N* (%)	575 (100)	66 (100)
Age M (SD)	24.06 (3.9)	24.61 (3.7)
Gender *N* (%)		
Male	156 (27.1)	18 (27.3)
Female	411 (71.5)	48 (72.7)
Not specified, diverse	8 (1.4)	–
Study period *N* (%)		
Preclinical	225 (39.2)	0 (0)
Clinical	349 (60.8)	66 (100)
Committed relationship *N* (%)		
Yes	339 (59.1)	42 (63.6)
No	235 (40.9)	24 (36.4)
Children *N* (%)		
Yes	33 (5.7)	5 (7.6)
No	541 (94.3)	61 (92.4)
Housing situation *N* (%)		
Alone	197 (34.5)	19 (29.2)
With others	374 (65.5)	46 (70.8)
Financial problems *N* (%)		
Yes	215 (37.4)	16 (24.2)
No	360 (62.6)	50 (75.8)

M, Mean; SD, Standard deviation.

#### Sub-study B

With *N* = 476 completed questionnaires from *N* = 1,418 enrolled medical students at the TUD, the response rate in 2020 was approx. 34%. *N* = 66 students who (1) participated in both study years (2020–2022) (2) provided complete information on all health behaviours and (3) provided a pseudonymisation code for identification, could be included in Sub-study B. In 2022, the study population was *M* = 24.61 years old (SD = 3.7) and 72.7% were female. The participants in Sub-study B are socio-demographically comparable to those in Sub-study A, except for differences in semester distribution (see Table 1).

#### Sub-study A—self-assessment of changes in health behaviour due to the COVID-19 pandemic | cross-sectional study

**Figure 2:**
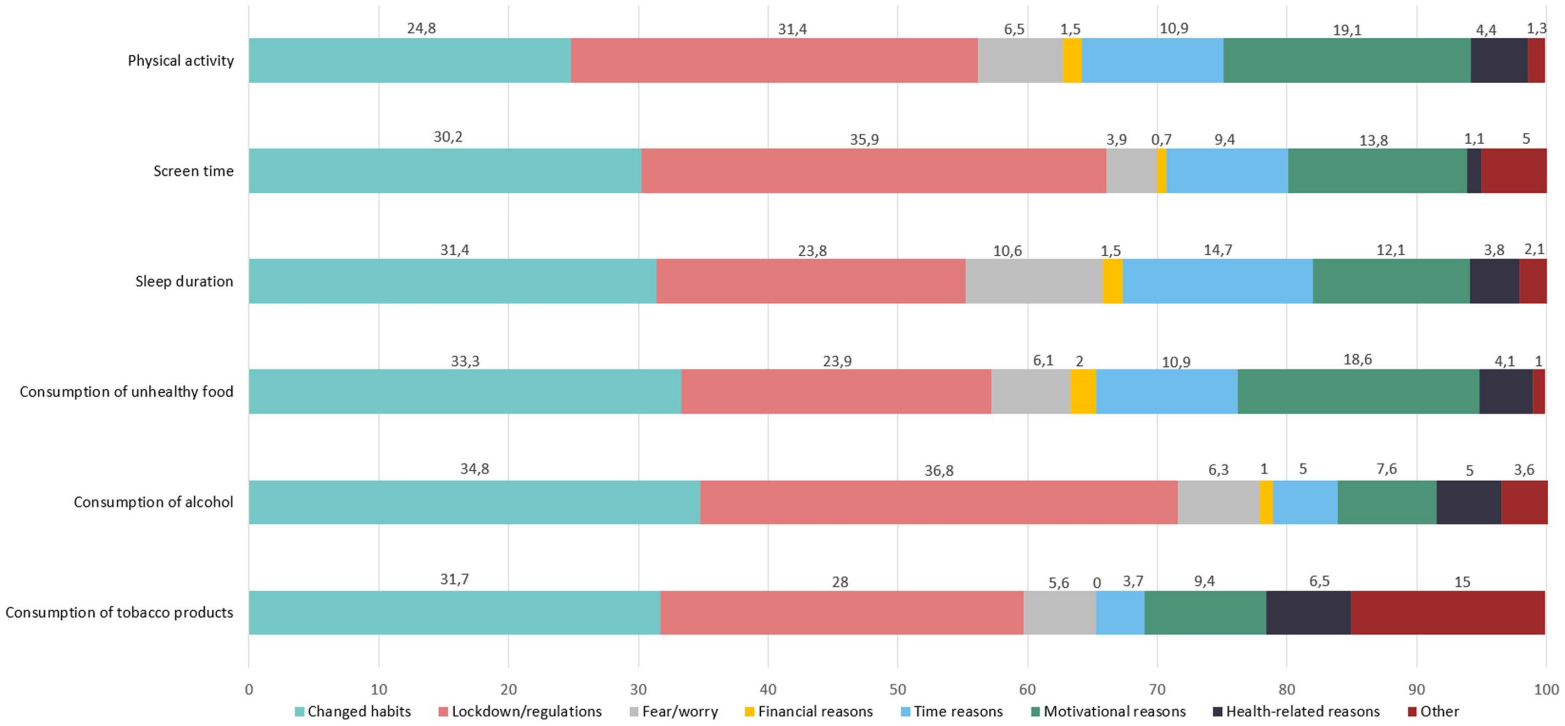
Reasons for changed health behaviour in % (multiple choice).

The self-assessment of the 6 different forms of health behaviour (physical activity, screen time, sleep duration, consumption of unhealthy foods, alcohol and of tobacco products) and their changes (decrease, no change, increase; single choice) are shown in Table 2. The respective reasons (changed habits, lockdown/regulations, fear/worries, financial reasons, time reasons, motivational reasons, health-related reasons, Other) are shown in Figure 2 (values see Supplementary material 3).

**Table 2:** Self-assessment of changes in health behaviour due to the COVID-19 pandemic (Single Choice).

Health behaviour	Change in health behaviour *N*, (%)			
	Decrease	No change	Increase	Total
Physical activity	271 (47.1)	221 (38.4)	83 (14.4)	575 (100)
Screen time	15 (2.6)	163 (28.4)	397 (69)	575 (100)
Sleep duration	55 (9.6)	414 (72.0)	106 (18.4)	575 (100)
Consumption of unhealthy foods	42 (7.3)	394 (68.5)	139 (24.2)	575 (100)
Consumption of alcohol	134 (23.4)	360 (66.3)	59 (10.3)	553 (100)
Consumption of tobacco products	42 (7.3)	500 (87.4)	30 (5.2)	572 (100)

#### Sub-study B—longitudinal analysis of health behaviour 2020–2022

##### Physical activity

In 2020, students exercised for an average of *M* = 124.5 min (SD = 92.7) per week at moderate intensity according to the definition of the WHO (see Supplementary material 2.1). In 2022, students exercised *M* = 191.0 min (SD = 154.0) at moderate intensity. The paired sample sign test showed a significant increase in the median from 120.0 min in 2020 to 130.0 min in 2022 (*z* = 2.375, *p* = 0.018).

In 2022, significantly (*p* < 0.001) more students (*N* = 33, 50%) exercised at least 150 min per week at moderate intensity than in 2020 (*N* = 17; 25.75%).

Among the students who showed a decrease in physical activity, the average decrease was *M* = −99.27 min, while for those who showed an increase, the average increase was *M* = +156.5 min (see Table 3).

**Table 3:** Longitudinal analysis of health behaviour change.

Health behaviour	Change			Total
	Decrease	No change	Increase	
Physical activity				
Longitudinal analysis *N* (%)	22 (33.3)	2 (3.0)	42 (63.6)	66 (100)
Average change in min per week (SD)	−99.27 (77.9)	–	+156.5 (133.9)	66.5 (167.1)
Screen time				
Longitudinal analysis *N* (%)	25 (37.9)	7 (10.6)	34 (51.5)	66 (100)
Average change in hours per day (SD)	−2.5 (1.8)	–	+2.6 (1.6)	0.41 (2.9)
Sleep duration				
Longitudinal analysis *N* (%)	25 (37.9)	16 (24.2)	25 (37.9)	66 (100)
Average change in hours per day (SD)	−1.04 (0.5)	–	+1.36 (1.1)	0.12 (1.3)
Consumption of unhealthy foods				
Longitudinal analysis *N* (%)	43 (65.2)	2 (3.0)	21 (31.8)	66 (100)
Average change in points per 3 months (SD)	−4.7 (5.8)	–	5.1 (4.7)	0.53 (7.02)
Consumption of alcohol				
Longitudinal analysis *N* (%)	30 (46.2)	9 (13.8)	26 (40.0)	66 (100)
Average change in number of drinks per year (SD)	−67.2 (87.4)	–	+110 (125.2)	12.9 (127.4)
Consumption of tobacco products				
Longitudinal analysis *N* (%)	4 (6.1)	59 (89.4)	3 (4.5)	66 (100)
Average change in points per month (SD)	−7.4 (9.4)	–	+5.4 (3.8)	−0.2 (3.0)

##### Screen time

In 2020, students spent an average of *M* = 4.9 h (SD = 2.2) in front of a screen for study/work reasons and *M* = 2.4 h (SD = 1.4) for leisure reasons. The total screen time (TST) in 2020 totalled *M* = 7.3 h (SD = 2.2).

In 2022, students spent *M* = 5.2 h (SD = 2.3) in front of a screen for study/work reasons and *M* = 2.6 h (SD = 1.5) for leisure reasons. The TST in 2022 was *M* = 7.71 h (SD = 2.4).

The Wilcoxon signed-rank test revealed no significant difference in screen time in 2020 vs. 2022 for both study/work (median: 4.5 h vs. 5.0 h, *z* = 1.026, *p* = 0.305) and leisure (median: 2.0 h vs. 2.0 h, *z* = 0.370, *p* = 0.711). The sign test for paired samples showed that the median difference in TST between 2020 (median: 7.0 h) and 2022 (median: 8.0 h) was not significant (*z* = 1.042, *p* = 0.298).

Among the students who showed a decrease in screen time (TST), the average decrease was *M* = −2.5 h, while for those who showed an increase, the average increase was *M* = +2.6 h (see Table 3).

##### Sleep duration

The students slept on average in 2020 *M* = 7.25 h per night (SD = 1.0) and in 2022 *M* = 7.37 h per night (SD = 1.1). The Wilcoxon signed-rank test showed that there was no significant difference (*z* = 0.000, *p* = 1.00) in the median (7.0 vs. 7.5 h) in sleep duration in 2020 vs. 2022.

In 2020, roughly as many students slept less than 7 h (*N* = 15, approx. 23%) as in 2022 (*N* = 14 students, approx. 24%, *p* = 1.00).

Among the students who showed a decrease in sleep duration, the average decrease was *M* = −1.04 h, while for those who showed an increase, the average increase was *M* = +1.36 h of sleep per night (see Table 3).

##### Consumption of unhealthy foods

On a scale of 0 to 100, the students achieved an average total score of *M* = 9.79 (SD = 7.7, range: 1.3–31.8) for the consumption of unhealthy foods in 2020 and a total score of *M* = 8.35 (SD = 7.6, range: 0.7–42.1) in 2022 [Reference: a value of 10 means that 1 group of unhealthy foods (e.g., fast food) was consumed twice a day in the last 3 months – scoring and food groups see Supplementary material 2.4].

The Wilcoxon signed-rank test showed that significantly more unhealthy foods were consumed in 2020 than in 2022 (median: 6.75 vs. 6.22, *z* = 2.625, *p* = 0.009).

Total scores for each food group [(A) fast food; (B) sweets, chocolate, cakes; (C) lemonade, juices, sugary drinks; (D) energy drinks] were calculated across all students (see Supplementary material 3). The score ranged from 0 to 1,650 where the maximum (1650) corresponds to the consumption of a food group by all students (*N* = 66) ≥5x/day (=25). The most frequently consumed food group across all students in 2020 and 2022 was (B) sweets, chocolate, cakes (373.3 and 349.4 points). While the consumption of all unhealthy food groups declined from 2020 to 2022, the biggest decrease was found for (D) energy drinks (37.8 vs. 6 points).

Among the students who showed a decrease in the consumption of unhealthy foods, the average decrease was *M* = −4.7 points, while for those who showed an increase, the average increase was *M* = 5.1 points (see Table 3).

##### Alcohol consumption

On average, students drank *M* = 103 alcoholic drinks in the last 12 months in 2020 (range: 0–455). In 2022, the average number of alcoholic drinks consumed in the last 12 months was *M* = 114 (range: 0–780). The Wilcoxon signed-rank test showed no significant difference in the median between alcohol consumption in 2020 and 2022 (median: 54.0 vs. 72.0; *z* = 0.401, *p* = 0.688).

The number of people who did not consume an alcoholic drink in 2020 was *N* = 11 people (16.7%), in 2022 *N* = 12 people (18.7%).

Among the students who showed a decrease in the consumption of alcohol, the average decrease was *M* = −67.2 drinks per year, while for those who showed an increase, the average increase was *M* = 110 drinks per year (see Table 3).

##### Consumption of tobacco products

Among all students, the average sum score (0–100) was *M* = 0.82 in 2020 and *M* = 0.62 in 2022. The median difference (0.0 vs. 0.0) was not significant (*z* = 0.0, *p* = 1.0) [Reference: a value of 7.6 means that 1 tobacco product (e.g., cigarettes) was consumed daily in the last 30 days – scoring see Supplementary material 2.6]. Among those who consumed tobacco products, the average sum score (0–100) was *M* = 10.9 in 2020 and *M* = 5.9 in 2022.

The number of those who consumed tobacco products in 2020 (*N* = 5 students, 7.5%) was not significantly different from the number who consumed tobacco products in 2022 (*N* = 7 students, 10%), *p* = 0.625.

Among the students who showed a decrease in the consumption of tobacco products, the average decrease was *M* = −7.4 points, while for those who showed an increase, the average increase was *M* = 5.4 points (see Table 3).

##### *Post hoc* analysis

As part of a *post hoc* analysis, the extent to which the individual changes in all health behaviours are related to each other was examined. The changes in the different health behaviours were widely independent of each other. Only the increase in physical activity showed a non-significant negative association with a decrease in the consumption of unhealthy foods (*r* = −0.150, *p* = 0.079) (see Supplementary material 4).

## Discussion

### Physical activity

In Sub-study B, physical activity among medical students increased significantly from 2020 to 2022, with 50% meeting the WHO guideline (14) of at least 150 min of moderat-intensity activity per week. Nearly two-thirds of students reported an increase, averaging about additional150 minutes weekly, while one-third recorded a decrease, averaging about 100 min. Few students reported no change. Although, high standard deviations (of up to 160 min overall) indicate high interindividual differences in the change of physical activity between 2020 to 2022. In Sub-study A, around 50% reported a reduction in their physical activity due to the pandemic – mainly due to lockdown and changed habits. This heterogeneous result, both in relation to the pandemic and in the longitudinal comparison between 2020 and 2022, is consistent with the existing literature to date. Whereas a systematic review reports a decrease in physical activity among students at the beginning of the pandemic (15) although later studies (16) cannot confirm a decrease. One of the reasons for this is that different forms of exercise (everyday activities, sport) have changed differently during the pandemic, depending on the respective daily routine. In contrast to reviews (2, 17) which show no differences in pandemic-related changes in physical activity based on sociodemographic factors such as age and gender, psychological, social and environmental factors seem to have an impact. Although these factors were not examined in this study, the findings provide the first description of the development of physical activity in a European student cohort from the start to the end of the pandemic.

### Screen time

Screen time for study/work, screen time for leisure reasons and conclusively total screen time increased slightly but non-significant from 2020 to 2022 among medical students. There were high interindividual variations ranging from a sharp decrease (−2.5 h) to a sharp increase (+2.6 h). In Sub-study A, around 70% of students stated that their screen time increased due to the pandemic and the measures, which is consistent with the current literature (18, 19). The increase can be primarily explained by the implemented online teaching and remote working during the pandemic. All students from Sub-study B were already in clinical semesters in 2022 where less online teaching took place and more attendance time in the clinic was required than in the pre-clinical semesters. This circumstance is likely to have contributed to the non-significant difference in screen time between 2020 and 2022. Moreover, although screen time increased during the pandemic (as shown in Sub-study A), the transition of online teaching and remote work back to in-person formats by the end of the pandemic may decreased screen time for medical students back to pre-pandemic levels. Up to date, there is little evidence on post-pandemic screen time, but existing findings (20) suggest a decrease compared to pandemic levels, which is in accordance with the present data.

### Sleep duration

Medical Students reported sleeping roughly the same amount in 2020 as in 2022 (4-week Ø 2020 *M* = 7.25 h per night; 2022 *M* = 7.37 h per night). In Sub-study A, approximately 70% of students reported that their sleep duration remained unchanged as a result of the COVID-19 pandemic. These results are congruent with the overall results of Sub-study B and an international meta-analysis (21) which concluded that the COVID-19 pandemic had no impact on the sleep duration of medical students. However, if we look at the study cohort from Sub-study B according to the categorical change (decrease, no change and increase), an increase or decrease can be seen in about 75% of students between 2020 and 2022. However, it is in the nature of the analysis that even small changes in the information provided by the students (for example, sleep duration 7.00 h in 2020 and 7.25 h in 2022) result in a change according to the categorisation. Furthermore, it seems plausible that the differing data from sub-studies A and B are not contradictory but instead reflect different time periods (e.g., the lockdown phase vs. typical behaviour in 2022). According to the AASM guideline, individuals should have at least 7 h of sleep per night (22). The fact that still every fifth medical students sleeps less than 7 h in 2022, highlights the before mentioned need (23) for interventions to promote healthy sleep habits.

### Consumption of unhealthy foods

The median consumption of unhealthy foods among medical students decreased slightly but significantly from 2020 to 2022. While the overall change has no pronounced clinical relevance, the average decrease (4.7 points) equates to about one fewer unhealthy food item per day, affecting approximately two-thirds of students. Although the exact consumption cannot be determined from the available data, all categories of unhealthy foods showed a decline, particularly energy drinks. The overall decrease from 2020 to 2022 contrasts with Sub-study A (almost 70% report no pandemic-related change in the consumption of unhealthy foods) and international systematic reviews (24, 25) which report an increase in unhealthy eating habits due to the pandemic. While the consumption of all unhealthy foods decreased in the present study, another systematic review (26) show heterogeneous results regarding the consumption of unhealthy foods (increase in sweets, decrease in fast food) due to the pandemic. Whether medical students eat more healthily than students of other disciplines is the subject of a contradictory debate (27, 28). Further studies are needed to provide a clearer picture of the dietary habits of medical students in Germany and to reconcile these contrasting findings.

### Alcohol consumption

Alcohol consumption among medical students did not differ significantly between 2020 and 2022, averaging about 1 drink per week in 2020 and 1.4 in 2022. The proportion of students abstaining from alcohol remained nearly unchanged (2020: 16.7%, 2022: 18.7%). However, some students reported a notable increase in consumption (Ø +110 drinks annually, _~_2 per week), while others reduced their intake by an average of 67 drinks (_~_1.3 per week). As this is a 12-month prevalence, the recorded rate provides a good reflection of alcohol consumption during the latter half of the pandemic. Similar to other health behaviours such as physical activity, a heterogeneous trend of change (either increase or decrease) is visible between 2020 and 2022. These findings are consistent with international studies, according to which the change in alcohol consumption due to the pandemic varied greatly (29, 30). In contrast, the proportion of students (_~_20%) abstaining from alcohol remained relatively stable, adhering to the WHO’s statement, that any level of alcohol consumption poses risks (31).

### Consumption of tobacco products

The prevalence of the consumption of tobacco products among medical students was not significantly different between 2020 (7.5%) and 2022 (10%). While a score of 7.6 reflects frequent use, medical students scored an average of 0.82 (2020) and 0.62 (2022), with cigarettes being the most commonly consumed product in 2022. Medical students in this cohort differ in this respect from a representative sample of the population aged 18–24, most of whom consume various tobacco products. The prevalence of cigarette smoking among medical students in 2022 (10%) is comparable to that of the general population in the same age group (approximately 10–13% in 2021) (32). In Sub-study A, almost 90% of students stated that their smoking behaviour had not changed as a result of the pandemic, which is comparable to medical students in other European countries (33, 34). Given that the 30-day prevalence of tobacco use among medical students can vary greatly depending on the survey period (e.g., exam period vs. non-exam period), it is important that future studies analyse longer time periods.

### Synopsis

While certain health behaviours, such as physical activity and consumption of unhealthy foods improved between 2020 and 2022, others like screen time and sleep duration as well as alcohol and tobacco consumption remained largely unchanged on average. Thereby, notable interindividual variations in health behaviour change among medical students were found. This is also reflected in the heterogeneous literature about health behaviour changes due to the pandemic. Partly, interindividual variations can be explained by different psychological, social and environmental situations students face. Furthermore, some students who participated in Sub-study A might not have been enrolled in medical school in 2020, suggesting a different living situation (e.g., attending school), which could inevitably be associated with different health behaviours. It cannot be conclusively determined whether differences between Sub-study A and Sub-study B are due to differences in content (reference to different time frames) and/or methodological differences (e.g., recall bias). The observed changes—significant increases in physical activity and reduced consumption of unhealthy foods as well as stable low rates of tobacco consumption point to an overall resilient and health-conscious cohort.

### Limitations

The data analysed in this study, was collected from a single German medical faculty, and the participation rate for Sub-study A was only 36%. In addition, only limited pseudonymised, matchable data was available for the longitudinal analysis in Sub-study B, which limits the generalisability of the results.

The study populations of Sub-study A and the pseudonymised sub-group of Sub-study B were easily comparable due to the given socio-demographic characteristics. The only unavoidable difference was that the students who had already been surveyed in 2020 were no longer represented in preclinical semesters 2 years later. Moreover, a change in semester phase might influence health behaviour, which is not directly accounted for in the present data.

There is data indicating that health literacy is higher among medical students than among non-medical students (13, 35). In case that increased health literacy cloud go along with more socially desirable responses regarding health behaviour, data could be distorted. Future studies should investigate whether socially desirable response behaviour to health questions is more common among medical students than students of other disciplines.

## Conclusion and outlook

To asses health behaviour change, this study employed a combined cross-sectional and longitudinal approach, which has been relatively less common in previous research. In particular, students stated that they were less physically active and spent more time in front of the screen due to the COVID-19 pandemic and the associated restrictions. The reasons given for this are in particular the lockdown and changed habits. The findings should inform the development of future preventive measures. Nevertheless, the longitudinal analysis showed that medical students were more physically active and consumed less unhealthy food after the end of the pandemic. Although a non-significant association between these two behaviours were found in the post-hoc analysis, in order to be able to speak of a trend, future studies must be conducted over a longer period of time with a higher number of cases. Overall, it can be stated that medical students in Dresden, Germany lead a relatively healthy lifestyle.

## Data Availability

The raw data supporting the conclusions of this article will be made available by the authors, without undue reservation. To ensure compliance with the data protection plan outlined in the ethics application, all individuals requiring access to the data must first be reported to the Ethics Committee of TU Dresden(No. EK 15012014). Upon notification and signing a data protection agreement, access to the data will be granted based on justified requests.
