# Supplementary material for: Changes in health behaviour of medical students during and after the COVID-19 pandemic—focus on physical activity, screen time, sleep duration, unhealthy foods, alcohol, and tobacco
Source: Front Public Health. 2025 Mar 24;13:1545295. doi: 10.3389/fpubh.2025.1545295 (PMC11973085; doi:10.3389/fpubh.2025.1545295)
Supplement: Supplementary file 1 [file Data_Sheet_1.pdf]

Supplementary 1 – Items Sub-study A

To assess the heath behaviour change due to the COVID-19 pandemic it was asked: "*To what extent has your health behaviour changed during the COVID-19 pandemic?*". The answer options were divided according to health behaviour in: "*Through the COVID-19 pandemic, I move/exercise (...), I spend (...) time in front of a screen (computer, tablet, mobile phone), I sleep (...), I eat (...) unhealthy foods, I drink (...) alcohol, I smoke (...)*. The students were able to choose between 5 answer options as to the extent to which the respective health behaviour has changed: *"significantly less than before", "slightly less than before", "not more or less than before", "a little more than before", "significantly more than before"*. For the following analysis, the response options were summarised as follows for the sake of clarity: "*decrease", "no change", "increase"*.

Through the Covid19 pandemic ...	significantly less than before	slightly less than before	not more or less than before	a little more than before	significantly more than before
I move/exercise …	☐	☐	☐	☐	☐
I spend ... time in front of a screen (computer, tablet, cell phone)	☐	☐	☐	☐	☐
I sleep …	☐	☐	☐	☐	☐
I eat … unhealthy foods	☐	☐	☐	☐	☐
I drink … alcohol	☐	☐	☐	☐	☐
I smoke…	☐	☐	☐	☐	☐

If the students indicated a change, they were given the opportunity to state the reasons why their health behaviour had changed. They were asked. "*Why has your health behaviour* [physical activity, screen time, sleep...] *changed through the COVID-19 pandemic?"*. The answer options (multiple answers possible) were: *changed habits, Lockdown/regulations, fear/worries, financial reasons, time reasons, motivational reasons, health-related reasons, other.*
