# Supplementary material for: Changes in health behaviour of medical students during and after the COVID-19 pandemic—focus on physical activity, screen time, sleep duration, unhealthy foods, alcohol, and tobacco
Source: Front Public Health. 2025 Mar 24;13:1545295. doi: 10.3389/fpubh.2025.1545295 (PMC11973085; doi:10.3389/fpubh.2025.1545295)
Supplement: Supplementary file 2 [file Data_Sheet_2.pdf]

Supplementary 2 – Items Sub-study B

The questions on health behaviour in 2020 and 2022 were asked and calculated as follows:

2.1 Physical activity

In both 2020 and 2022, students were asked about moderate-intensity exercise, with the question in 2020 being:

1) *On average, how many days a week are you so active physically that you sweat or get out of your breath?"* | Answer option: … *days*

2) *On average, how long are you physically active on these days when your physical activity makes you sweat or out of breath?"* | Answer options: *less than 10 minutes, 10 to less than 30 minutes, 30 to less than 60 minutes, more than 60 minutes*

A total score in minutes was calculated by multiplying the number of days by the time specified. For "*less than 10 minutes*" 5 minutes were used, for *"10 to less than 30 minutes"* 15 minutes, for "*30 to less than 60 minutes*" 45 minutes and for "*more than 60 minutes*" 60 minutes. The questions were based on the then current guideline of the WHO on physical activity (36) the items as well as the calculation were adopted from the DEGS study (37).

Towards the end of 2020, the WHO published new guidelines on physical activity (14), which formed the basis of the items created in 2022. Moderate-intensity exercise was defined in in the survey (according to WHO) as "Moderate intensity: breathing is somewhat accelerated, you may sweat slightly, you can still talk but not sing during exercise" and asked

1) *"How much physical activity of moderate-intensity do you do in a typical week?*" | Answer option: *in minutes per week*.

The average physical activity with moderate intensity in minutes per week for 2020 and 2022 were compared using a paired sample sign test.

2.2 Screen time

The questions on screen time were almost identical in 2020 and 2022.

In 2020 it was asked:

How much time during a regular day do you spend at your computer/tablet pc/cell phone...

... for your studies/work? | Answer option: Ca. …hours

... during your leisure time (z.B. browsing/social media/games/videos)? | Answer option: Ca. …hours

In 2022 it was asked:

On average, how much time do you spend daily on the computer/tablet PC/mobile phone...

...for study/work? | Answer option: Approx. …hours

...for leisure (e.g., browsing/social media/games/videos)? | Answer option: Approx. …hours

The screen time given for study/work and leisure time was totalled and a new variable, total screen time (TST), was calculated. A comparison of screen time (study/work, leisure time, TST) in hours per day from 2020 and 2022 was carried out using the Wilcoxon signed-rank test.

2.3 Sleep duration

The duration of sleep was asked identically in 2020 and 2022 via the corresponding item from the PSQI (38) *"During the past month, how many hours of actual sleep did you get at night? (This may be different than the number of hours you spend in bed.)"* | Answer option: *in hours.*

A group comparison of the average sleep duration in hours per night from 2020 and 2022 was realised with a paired sample sign test.

2.4 Consumption of unhealthy foods

Food consumption was recorded identically in 2020 and 2022. The students had to answer how often they had consumed the following food groups in the last 3 months: (A) fast food; (B) sweets, chocolate, cakes, etc.; (C) lemonade, juices, sugared soft drinks; (D) energy drinks. The answer options were: ≥5x/day, 2-4x/day, once a day, several times a week, once a week, 1-3x/month, never.

	≥5 times a day	2-4 times a day	once a day	several times a week	Once a week	1-3 times a month	never
(A) Fast food	25 (450)	15	5	2.83	0.72	0.33	0
(B) Sweets, chocolate, cakes, etc.	25	15	5	2.83	0.72	0.33	0
(C) Lemonade, juices, sugared soft drinks	25	15	5	2.83	0.72	0.33	0
(D) Energy drinks	25	15	5	2.83	0.72	0.33	0

For the scoring, 3 months were equated with 90 days and the following points were given for the respective frequencies in descending order: (≥ 5x/day = 450), (, 2-4x/day = 270), (once a day = 90), (several times a week = 51), (once a week = 13), (1-3 times a month = 6), (never = 0). For every student, an overall score was calculated across all food groups, which ranged from 0 to 1800. For better understanding, this score was standardized to a scale of 0 – 100 whereas an unstandardised score of 450 (≥5x/day consumption of a unhealthy food group for 90 days) equals a standardized score of 25. A comparison of the total scores from 2020 and 2022 was carried out via a Wilcoxon signed-rank test. As a reference, a standardised score of 10 indicates that 1 group of unhealthy foods was consumed 2 times a day or that 2 groups of unhealthy foods were consumed once a day.

In addition, total scores for each food category were calculated across all students on a scale from 0 to 1650. The maximum (1650) corresponds to the consumption of a food group by all students (N=66) ≥5x/day (=25).

2.5 Alcohol consumption

In 2020, students were asked:

1) *" How often during the past 12 months did you have an alcoholic drink (e.g. a glass of wine, beer, mixed drinks, schnaps, liqueur)?"* | Answer options: *never, once a month or less, 2-4x/month, 2-3x/week, >=4x/week*

2) *"If you drink alcohol, how many alcoholic drinks do you typically drink in a day?"* | Answer options: *1-2, 3-4, 5-6, 7-8, 9 or more*

In 2022, the questions were slighty modified:

1) *"How often during the past 12 months did you have an alcoholic drink? (an alcoholic beverage = a small bottle of beer (0.33 l), a small glass of wine (0.125 l), a glass of sparkling wine (0.1 l), a shot of distilled spirits (e.g. tequila, vodka) (4 cl) or a bottle of alcopops/Ready to Drink (0.33 l))."* | Answer options: *never, once a month or less, 2-4x/month, 2-3x/week, >=4x/week*

2) *"If you drink alcohol, how many alcoholic drinks do you typically drink in a day?"* | Answer options: *1,2, 3,4, 5,6,7,8, 9 or more*

A total score for 12 months (=52 weeks) was calculated by multiplying the answers to question 1 and question 2. For this purpose, the following values were assumed for the answer options for question 1 (2020, 2022): (*never* = 0)*, (once a month or less* = 6), *(2-4x/month* = 36), *(2-3x/week* = 130), *(>=4x/week* = 208). The frequencies from question 1 were then multiplied by the number of drinks (=answers to question 2), whereby mean values were formed for question 2 from 2020 (1.5; 3.5; 5.5; 7.5; 9) and the number of drinks in 2022 was given from 1-9. The total score then represented the number of alcoholic drinks per student in the past 12 months. The total scores from 2020 and 2022 were compared using a Wilcoxon signed-rank test.

2.6 Consumption of tobacco products

The consumption of tobacco products (traditional and alternative tobacco products) in 2020 and 2022 was recorded identically. Students were asked: "During the past 30 days how often did you consume..." (Products: Cigarettes, tobacco-free hookah/shisha, hookah/shisha with tobacco, nicotine-free e-cigarette, nicotine-containing e-cigarette, heated tobacco products (e.g. IQOS), snus. The possible answers were: never, 1-3 times, once a week, several times a week, daily.

	never	1 - 3 times	once a week	several times a week	daily
Cigarettes	0	0.95	1.90	7.62	14.28
Tobacco-free hookah/shisha	0	0.95	1.90	7.62	14.28
Hookah/shisha with tobacco	0	0.95	1.90	7.62	14.28
Nicotine-free e-cigarettes	0	0.95	1.90	7.62	14.28
Nicotine-containing e-cigarettes	0	0.95	1.90	7.62	14.28
Heated tobacco products (e.g. IQOS)	0	0.95	1.90	7.62	14.28
Snus	0	0.95	1.90	7.62	14.28

For each student, an unstandardised total score (0-210) was calculated for the consumption of all tobacco products (N = 7) within the last 30 days, in which the answer options (never = 0), (1-3 times = 2), (once a week = 4), (several times a week = 16), (daily = 30) were multiplied by the number of respective tobacco products (0-7). For better understanding, this score was converted to a standardized scale of 0 – 100, whereas an unstandardised score of 30 (daily consumption of a tobacco product) equals a standardized score of 14.28. A value of 7.62 means that one type of tobacco product was consumed several times a week.

The individual total score of the tobacco products consumed in 2020 and 2022 was compared using a Wilcoxon signed-rank test.

In addition, total values were calculated for each product category across all students on a scale from 0 to 1980. The maximum (1980) corresponds to the consumption of a product by all students (N=66) each day (=30).
