# Supplementary material for: Changes in health behaviour of medical students during and after the COVID-19 pandemic—focus on physical activity, screen time, sleep duration, unhealthy foods, alcohol, and tobacco
Source: Front Public Health. 2025 Mar 24;13:1545295. doi: 10.3389/fpubh.2025.1545295 (PMC11973085; doi:10.3389/fpubh.2025.1545295)
Supplement: Supplementary file 3 [file Data_Sheet_3.pdf]

Supplementary 3 Sum values of the unhealthy food groups across all students in 2020 | 2022

Food group / year	2020	2022	Change 2020 vs. 2022, in points
(A) Fast food	84.7	73.9	-10.8
(B) Sweets, chocolate, cake	373.3	349.4	-23.9
(C) Lemonade, juices, sugary drinks	149.9	133	-16.9
(D) Energy drinks	37.8	6	-31.8
