# Supplementary material for: Changes in health behaviour of medical students during and after the COVID-19 pandemic—focus on physical activity, screen time, sleep duration, unhealthy foods, alcohol, and tobacco
Source: Front Public Health. 2025 Mar 24;13:1545295. doi: 10.3389/fpubh.2025.1545295 (PMC11973085; doi:10.3389/fpubh.2025.1545295)
Supplement: Supplementary file 4 [file Data_Sheet_4.pdf]

Supplementary 4 Kendal's tau-B correlation matrix of the changes in health behaviour

Change in	Physical activity	Screen time	Sleep duration	Consumption of unhealthy foods	Alcohol consumption	Tobacco Consumption
Physical activity		-.079 p=0.367	.077 p=0.388	-.150 p=0.079	.124 p=0.155	-.001 p=0.992
Screen time	-.079 p=0.367		-0.59 p=0.517	.007 p=0.933	.009 p=0.918	0.17 p=0.867
Sleep duration	.077 p=0.388	-0.59 p=0.517		-0.069 p=0.439	.055 p=0.546	-.051 p=0.629
Consumption of unhealthy foods	-.150 p=0.079	.007 p=0.933	-0.069 p=0.439		-.020 p=0.820	-.030 p=0.763
Alcohol consumption	.124 p=0.155	.009 p=0.918	.055 p=0.546	-.020 p=0.820		.085 p=0.409
Tobacco consumption	-.001 p=0.992	0.17 p=0.867	-.051 p=0.629	-.030 p=0.763	.085 p=0.409	
